# Prediction and Characterization of Missing Proteomic Data in *Desulfovibrio vulgaris*

**DOI:** 10.1155/2011/780973

**Published:** 2011-05-04

**Authors:** Feng Li, Lei Nie, Gang Wu, Jianjun Qiao, Weiwen Zhang

**Affiliations:** ^1^Division of Biometrics II, Office of Biometrics/OTS/CDER/FDA, Silver Spring, MD 20993-0002, USA; ^2^Division of Biometrics IV, Office of Biometrics/OTS/CDER/FDA, Silver Spring, MD 20993-0002, USA; ^3^Department of Biological Sciences, University of Maryland at Baltimore County, Baltimore, MD 21250, USA; ^4^School of Chemical Engineering and Technology, Tianjin University, Tianjin 300072, China

## Abstract

Proteomic datasets are often incomplete due to identification range and sensitivity issues. It becomes important to develop methodologies to estimate missing proteomic data, allowing better interpretation of proteomic datasets and metabolic mechanisms underlying complex biological systems. In this study, we applied an artificial neural network to approximate the relationships between cognate transcriptomic and proteomic datasets of *Desulfovibrio vulgaris*, and to predict protein abundance for the proteins not experimentally detected, based on several relevant predictors, such as mRNA abundance, cellular role and triple codon counts. The results showed that the coefficients of determination for the trained neural network models ranged from 0.47 to 0.68, providing better modeling than several previous regression models. The validity of the trained neural network model was evaluated using biological information (i.e. operons). To seek understanding of mechanisms causing missing proteomic data, we used a multivariate logistic regression analysis and the result suggested that some key factors, such as protein instability index, aliphatic index, mRNA abundance, effective number of codons (*N*_*c*_) and codon adaptation index (CAI) values may be ascribed to whether a given expressed protein can be detected. In addition, we demonstrated that biological interpretation can be improved by use of imputed proteomic datasets.

## 1. Introduction

Application of various experimental “omics” tools has enhanced our understanding of complex biological systems in the past decade [1–8]. However, due to technical limitations of these high throughput technologies and constraints on experimental design, most of these high-throughput “omics” datasets still suffer from significant missing values. The incomplete data available has impeded scientists from extracting correct information regarding cell metabolism. To address the issue, attempts to apply computational tools to impute missing values in various high throughput “omics” datasets have been made [9–11]. The most successful examples of such efforts were for missing transcriptomic data [12]. 

Compared with transcriptomic dataset, proteomic datasets suffer even more serious missing data since its identification range and sensitivity are still not fully comparable with transcriptomic measurements [13]. In particular, when partial proteomics dataset was used in various integrated “omics” studies, the undetected proteins were simply assigned a “zero” value and were excluded from relationship modeling, which could bias any conclusion resulted from the integrated studies [14]. To overcome this problem, several methods have been adapted from the estimation of missing values in transcriptomic data to estimate the missing proteomics values by using the available measurements from other proteins, such as the *k* nearest neighbor and Bayesian Principal Component Analysis (BPCA) methods for imputing missing proteomic values in gel-based proteomics dataset [15, 16], and by integrating the GO (Gene Ontology) information into the proteomic data imputation [17]. Based on the assumption that there exists meaningful correlation between two types of datasets [14], in recent years we have developed the Zero-inflated *Poisson* (ZIP) linear regression model [18] and a stochastic Gradient Boosted Trees (GBT) nonlinear model [19] to uncover possible relationships between transcriptomic and proteomic data and to predict protein abundance for the proteins not experimentally detected.

As part of a continuous effort to develop statistical tools for missing value imputation, we in the study applied an artificial neural network (ANN) approach to predict abundance for experimentally undetected proteins for several cognate transcriptomic and proteomic datasets of sulfate reducing bacterium *Desulfovibrio vulgaris* [20–23]. The advantages of artificial neural networks include ability to tackle complex relationships and to deal with noisy data with missing values, and its capability of generalization [24, 25]. In the approach, a multilayer perceptron (MLP) with sigmoid activation function in the single hidden layer was used to capture the nonlinear relationship between transcriptomic data and protein abundance. The top ten relevant predictors for protein abundance identified in a previous study [19] were used as inputs to the MLP for training the output of the MLP to approximate the corresponding protein abundance of each gene. The trained MLP was validated using biological information. In addition, by using a multiple logistic regression analysis, we quantified the contribution of some sequence-based factors, such as mRNA abundance, protein instability index, gene length, “effective number of codons” (*N*_*c*_), and CAI (Codon Adaptation Index) values to missing proteomic values. We also demonstrated that biological interpretation can be improved by using the imputed proteomic datasets.

## 2. Materials and Methods

### 2.1. Datasets

Two datasets from *Desulfovibrio vulgaris* were analyzed in this study. The datasets covers the same species, although the experimental conditions are different and they were obtained by two independent studies [19–23]. The raw intensity values from both datasets were normalized using a quantile normalization in an R package (caret) available through the *R* project (http://www.r-project.org/). Absolute fluorescence intensity and peptide hits were used as abundance for transcriptomic and proteomic data, respectively [26]. These datasets, labeled Dataset 1 and Dataset 2, will be used throughout the article.

### 2.2. Genome Information

Gene annotated attributes, such as sequence length, protein length, molecular weight, GC content, triple codon counts, and cellular functional categories of all genes in the *D. vulgaris* genome were downloaded from the comprehensive microbial resource (CMR) of TIGR (http://cmr.tigr.org/) [22]. To define operons, we used the distance-only approach, a relatively low threshold, in this study to cover more of the possible operons. The genes that are transcribed in the same direction and are with intergenic region less than 15 base pairs were defined as one operon [19].

### 2.3. Construction of Relationship between Proteomics and mRNA through Artificial Neural Network

We trained an artificial neural network to learn a particular function by adjusting the values of the weights between neurons. It has been shown that a multilayer perceptron with a single hidden layer can approximate any continuous function to arbitrary precision by increasing the number of neurons in the hidden layer [27–29]. The weights connecting the input signals as well as the number of neurons can be determined by minimizing the sum of the squared difference between the network outputs and target outputs for each set of input signals. Nonlinear optimization methods in combination with a backpropagation algorithm [30] were implemented to find the optimal weights. The inputs to the network and the targets were usually rescaled to [−1, 1]. The outputs of the network were then rescaled back to approximate the targets in original scale. In this work, we trained a multilayer perceptron to learn the nonlinear relationship between transcriptomic data and proteomic data. The inputs were the transcriptomic data and the target was the protein abundance. 

A feed-forward network with a hidden layer can approximate any continuous function with arbitrary precision, which provides ANN the power to deal with nonlinear relationship, also open a door to overfitting if too many neurons are used in the hidden layer. To address the possible overfitting, we implemented a cross-validation to prevent overfitting. Through this method the data was partitioned in *K* equal parts where *K* − 1 sets were used to train the model and the other set was used to calculate prediction errors [31]. This was repeated *K* times, hence *K* prediction errors values were computed one at every fold. An average and standard deviation can be extracted to select the most representative model for future prediction. Cross-validation was generally effective to prevent overfitting. Beside cross-validation to prevent overfitting, we also used some statistical tools to diagnose the selected model. Once the best model has been selected, based on cross-validation, the model was evaluated based on its coefficient of determination (*R* ^2^). This coefficient represents the variation explained by the model. Furthermore, to alternatively assess the goodness of the model we studied the predictions of small sets of genes which are grouped based on operon information. In order to describe the variation within a data set, such as “molar abundance” of proteins within one operon, we computed the coefficient of variation (CV) for each set of proteins. The CV is defined as the ratio of the standard deviation and the mean of the “molar abundance” for a set of proteins [18, 19, 32] where the calculation of CV score is independent of the sample size. These coefficients of variations were computed for all operons and compared to a distribution of permuted CVs where permutation of genes was performed.

### 2.4. Analysis of the Factors Ascribed to Missing Protein Measurements

 For DNA and protein sequence features, the *BioPerl* module was used to batch query and parse results from the ExPASy ProtParam program, returning “AliphaticIndex” and “InstabilityIndex”. CodonW was used to perform correspondence analysis of both codon and amino acid usages, returning the first four axes representing the major trends (AA_axis1–4 for amino acid usage, CR_axis1–4 for Relative Synonymous Codon Usage). The frequency of each base at the third codon position (synonymous site, designated as A3s, T3s, C3s, and G3s), as well as two additional measures on protein property such as Grand Average of Hydropathy (GRAVY) and AROMO, were also computed with CodonW. The Codon Adaptation Index (CAI) was computed with an in-house perl script using *D. vulgaris* ribosomal protein genes as reference for highly expressed genes. The calculation and analysis of these features were described in several of our previous works [33–35]. 

A logistic regression model was used to assess how each of the features attributes to whether a given protein will be detected [36, 37]. Assume the features for a gene *i* are *x*_1*i*_,…, *x*_*ki*_. In logistic regression, the logit, natural log of the odds, of the unknown probability, *p*_*i*_, that the protein is not detected is modeled as a linear function of *x*_1*i*_,…, *x*_*ki*_:

(1) $$\log\;\;\text{it}\left(p_{i}\right)=\ln\;\left(\frac{p_{i}}{1-p_{i}}\right)=\beta_{0}+\beta_{1}x_{1i}+\cdots +\beta_{k}x_{ki}.$$

The model is equivalent to

(2) $$p_{i}=\frac{\exp\;\left(\beta_{0}+\beta_{1}x_{1i}+\cdots +\beta_{k}x_{ki}\right)}{\exp\;\left(\beta_{0}+\beta_{1}x_{1i}+\cdots +\beta_{k}x_{ki}\right)+1}.$$

The unknown coefficients are usually estimated by maximum likelihood using a method common to all generalized linear models [35]. In the model, the parameter *β*_*j*_ is the additive effect on the log of the odds for a unit change in the *j*th explanatory feature variable. That is, one unit change of feature *j*, that is, *x*_*ji*_ indicates the odds changes *e* ^*β*_*j*_^ times. Positive *β*_*j*_ indicated that the probability that the protein will not be detected is increased with increasing the *j*th feature. By using a logistic regression analysis, the effects of relevant features on whether a protein will be detected were quantified and can be compared. And the fitted model can also be used to predict the chance that any other proteins will not be detected. 

## 3. Results and Discussion

### 3.1. Determine the Variable Importance and Partial Variable Dependence

**Figure 1:**
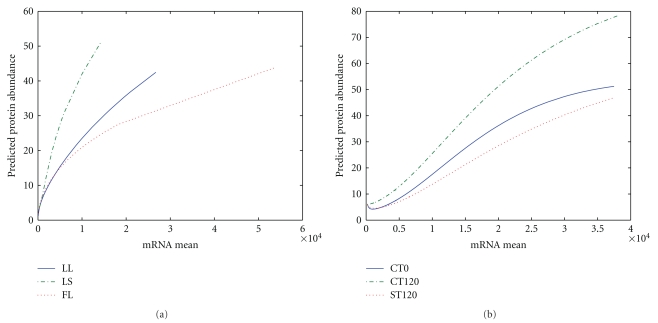
Partial dependency plots. Predicted protein abundance for given values of mRNA controlling the other predictors at their mean values. (a) Dataset 1; (b) dataset 2.

**Figure 2:**
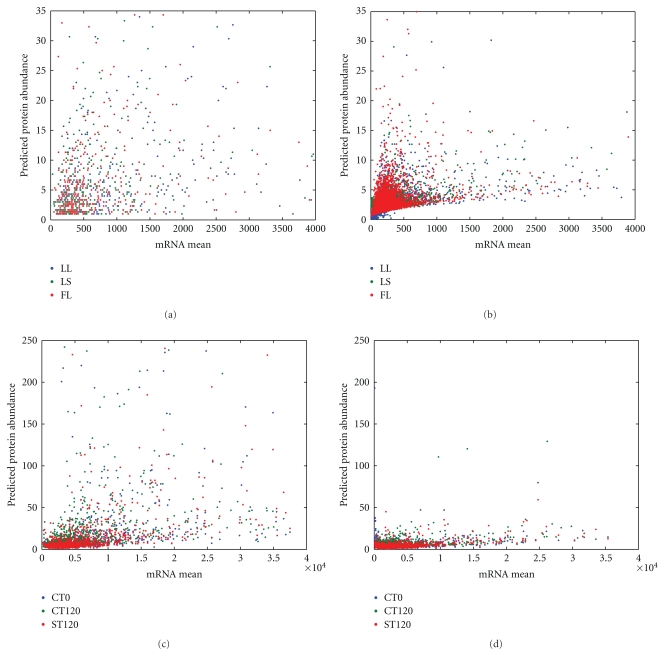
Prediction plot for proteins. Scatter plots of predicted protein abundance versus mRNA for proteins detected and undetected. (a) Plot for genes with detected proteins for dataset 1; (b) plot for genes with undetected proteins for dataset 1; (c) plot for genes with detected proteins for dataset 2; (d) plot for genes with undetected proteins for dataset 2.

In a previous study, we used the gradient boosted trees (GBT) model to measure the contribution of each of the 70 variables input variables to the RNA-protein correlation, and the 10 top-ranked variables were identified to be mRNA expression level, cellular role, and several triple codon usages for the *D. vulgaris* datasets we used (See Table  1 of Supplmentary Material available online at doi: 10.1155/2011/780973) [19]. In this study, we used the same top ten predictors as inputs to the artificial neural network approach. The datasets used are consistent with previous work for comparison. The results showed that for all datasets, we observe a smooth relationship between protein abundance and the abundance of corresponding mRNA controlling the other predictors at their mean values for each growth condition. The partial dependence between protein and mRNA abundance tends to be smoother when the abundance of mRNAs are in the high range (Figure 1).  Figure 2 shows the scatter plots of predicated protein abundance versus mRNA for proteins detected and undetected for datasets 1 and dataset 2, respectively. For both datasets, under each growth condition, there are much fewer genes with very high mRNA or protein abundance. Those proteins undetected are mostly with very low predicted protein abundance and observed mRNA abundance as shown in Figures 2(b) and 2(d) for dataset 1 and dataset 2, respectively. The proteins undetected have much smaller variance in predicted values especially for dataset 2 as shown in Figure 2(d). In comparison, those detected proteins have a larger range of protein abundance and are of very high variability. 

In the previous work of using GBT model to uncover the nonlinear relationship between transcriptomic and proteomic datasets, an interesting pattern, a plateau effect, was observed in the partial dependence plot of mRNA abundance versus protein abundance for high values of mRNA. This is potentially due to the fact that protein abundance data is sparse, with high variance, where the tree models do not generate splits among the predictors [19]. In the neural network model, the output of an MLP is an inherent smooth function of the network inputs, hence a relatively smooth prediction is always guaranteed. However, we can still see the similar pattern that the curve becomes flat when the variance for the mRNA abundance is high. For example, in the region of high mRNA values there are a small number of genes/peptides whose protein values ranges from (0, 40) for Dataset 1 and (0, 500) for Dataset 2. This could reflect problems with the sensitivity of the current proteomic technologies for high abundant proteins. 

### 3.2. Construction of the Nonlinear Artificial Neural Network Model

A feed-forward network with a single hidden layer was trained for each condition for both datasets with mRNA abundance and other predictors as inputs and protein abundance as target. The neural networks were trained using the functions in the neural network toolbox provided in standard software Matlab (7.0.1). The activation function for the neurons in the hidden layer was the hyperbolic tangent function, which is given by

(3) $$f\left(v\right)=\frac{1-\exp\;\;\left(-v\right)}{1+\exp\;\;\left(-v\right)}.$$

The activation function of the output neuron is a pure linear function, which is the weighted sum of the outputs from the neurons of the hidden layer. The optimal number of neurons in the hidden layer and the optimal weights were determined using 10-fold cross-validation for training a neural network. For each dataset under each experimental condition, the networks with the same inputs but with different number of neurons (from 5 to 12) in the hidden layer were each trained using cross-validation to learn the relationship between the mRNA features and protein abundance. The optimal number of neurons in the layer was determined based on the one generating the minimum averaged validation error. 

 Both transcriptomic and proteomic data were log-transformed. For dataset 1, the genes with no missing value for three out of four replicates were selected for model training. The numbers of genes used for training were 354, 312, and 308 for the FL, LL, and LS conditions, respectively. For each growth condition, the inputs to the multilayer perceptron were the top ten predictors identified previously [19] (See Table  1 at Supplementary Material). The optimal network had 6, 6, and 9 neurons in the hidden layer for FL, LL, and LS conditions, respectively. Coefficients of determinations *R* ^2^ were 0.47, 0.52, and 0.51 for FL, LL, LS conditions, respectively. For dataset 2, a total of 986, 1001, and 986 genes with no missing protein measurements were used for model training for CT0, CT120, and ST120 conditions, respectively. For each of the conditions, the inputs to the multilayer perceptron were the top ten predictors identified previously [19] (See Table  1 at Supplementary material). The optimal network had 8, 7, and 6 neurons in the hidden layer for CT0, CT120, and ST120 conditions, respectively. Coefficients of determination *R* ^2^ were 0.65, 0.62, and 0.68 for CT0, CT120, and ST120 conditions, respectively.

### 3.3. Validation of the Prediction through the Nonlinear Artificial Neural Network Model

The validation of the model prediction was conducted through the calculation of coefficient of variation (CV) across conditions for every operon of *D. vulgaris* as described before [18, 19]. The genes in the operons are considered to have less dispersion than any random set of genes in terms of their gene expression and/or protein abundance, because of their intrinsic biological relationship. With relatively low threshold for operon identification as described in Section 2, a total of 609 operons were identified in *D. vulgaris*, which ranged from 2 to 13 genes per operon. To compare these CV values, we have used a permutation test in the following manner: a CV is computed from the predicted protein values for a permuted number of genes. This step is repeated 2000 times through resampling of genes without replacement. The number of genes randomly selected is equal to the size of the gene group to be compared to. For dataset 1, 82% to 89% of the operon groups had smaller CV values than the permuted groups, and for dataset 2, 79% to 89% of the operon groups had smaller CV values than the permuted, demonstrating in general good performance of the model prediction. The validation results are summarized in Table 1. 

To further demonstrate how the current measurement of dispersion (CV) compares with the distribution of the permuted coefficients of variation, we also calculated the percentile score for all coefficients of variations, based on the biological knowledge that genes from the same operon should be less dispersed than permuted sets of genes and the percentile scores are thus expected to be low. The results showed that even though the calculated CV for most groups was less than the mean CV value for permuted sets of genes, the percentile scores shows that only a small collection of these groups fall within a percentile less than 0.20. The percentage of groups with percentile scores less than 0.2 is 43% to 57%, and 57% to 61% for dataset 1 and dataset 2, respectively.

### 3.4. Comparison of Artificial Neural Network to Previous Methods (GBT and ZIP)

Similar to the GBT method, the artificial neural network method aims to identify an unknown and potentially nonlinear relationship between proteomics and mRNA abundance data, which distinguish them from the ZIP method, which carries zero-inflated linear assumptions [18]. Another key difference is that GBT and neural network methods utilized additional sequence based information, such as cellular role, gene length, amino acid usage, and codon usage known to be important for translation efficiency to model the relationship between transcriptomic and proteomic dataset, while the ZIP method establishes the relationship between proteomic and mRNA solely based on experimental transcriptomic and proteomic dataset. 

Some improvements were observed in terms of prediction validation by the neural network model in comparison to the GBT model. The percentage of operon groups with CV smaller than permuted CV was 82% to 89% for dataset 1 in comparison to 75% to 79% for the GBT model. The percentage of operon groups with CV smaller than permuted CV was 79% to 89% for dataset 2 by the neural network model in comparison to 75% to 82% by the GBT model. For dataset 1, the percentage of groups with percentile scores less than 0.2 is 43% to 57% in the neural network model in comparison to 36% to 50% in the GBT model. For dataset 2, the percentage of groups with percentile scores less than 0.2 is 57% to 61% in the neural network model in comparison to 32% to 54% in the GBT model. The coefficient of determination was 0.47 to 0.58 for dataset 1 and 0.62 to 0.68 for dataset 2 in the neural network model in comparison to 0.39 to 0.58 for both datasets in the GBT model, demonstrating the artificial neural network model could be a good alternative in analyzing the missing proteomic values.

### 3.5. Analysis of Factors Ascribed to Missing Protein Measurements

Through the neural network model, the majority of the proteins that were experimentally undetected were predicted to have protein abundance with greater than 1.0 peptide hit. This thus tempted for us to look into the possible factors which may be ascribed to the missing protein values. To do so, we collected some of the protein or mRNA/DNA sequence-based features which may affect protein detectability for use along with experimental transcriptomic data in a logistic regression procedure to determine their relative contribution to detectability of proteins in the *D. vulagris* genome. The features/measurements we used were from the following several categories: (i) features related to gene expressivity: experimental mRNA abundance data, *N*_*c*_ and CAI values [33, 34], codon usage such as CR_axis1-CR_axis4, T3s, C3s, A3s, G3s, GC3s, and GC content [35]; (ii) features related to protein and RNA stabilities: protein instability index and AliphaticIndex, and minimal free energy (MFE) of RNA [35]; (iii) features related to protein solubility: protein solubility indices such as Grand Average of Hydropathy (GRAVY) [33]; (iv) amino acid usage such as AA_axis1-AA_axis4, and others such as gene length [35]. The logistic regression procedure in the SAS software package was used to model the probability of whether a protein will be experimentally detected given the features and measurements for the gene/protein. The stepwise selection method was used to select those most influential factors for indicating missing proteomic value. Those highly correlated features were removed automatically. The 0.05 significance threshold was used for the stepwise selection. The mRNA abundance and gene length values were both log2 transformed before being used in the procedure. The protein stability index, aliphaticIndex, and *N*_*c*_ values were divided by 10 and the CAI, N_MFE, GRAVY, AA_axis1-AA_axis4, and CR_axis1-CR_axis4 were all multiplied by 10 so that they scaled to 1. This standardization will not change the results, but make illustration of the results easier.

Tables 2 and 3 present the odds ratio of each selected factor for dataset 1 and dataset 2, respectively. The results showed that some of the features are highly correlated with probability of missing proteomic values. For dataset 1, seven factors were indentified consistently by the regression model. Among the features that are positively correlated with probability of missing proteomic values (i.e., when the features/measurement increase), protein instability index which provides an estimate of the stability of a given protein in a test tube [38] was found as the most significant factor for all three growth conditions, and aliphaticIndex which is regarded as a positive factor for the increase of thermostability of globular proteins [39], was found significant for growth conditions LL and FL, but not for condition LS. In addition, AA_axis2 and CR_axis3 correlated most strongly with GRAVY and G3s, respectively, and they were also found significant in all three growth conditions, while the N_MFE was only significant for LS condition (Table 2). For dataset 2, the features that are kept in the regression model are more the same for the three growth conditions in dataset 1, suggesting that these factors could be universal across experimental platforms. Six features that are significant for dataset 1 are also found to be critical for all the conditions and affect the probability in the same direction for dataset 2 include mRNA abundance, protein instability index, gene length, *N*_*c*_, CAI, and AA_axis2 corresponding to GRAVY (Table 3). In general, the analysis suggested that those proteins with smaller mRNA expression values, smaller *N*_*c*_ value, higher protein instability index, or high value of aliphaticIndex, have a greater probability to have missing proteomic values in the *D. vulgaris* datasets. Based on this preliminary analysis, it may be possible to apply a logistic regression model to predict whether a given protein will or will not be detected by choosing a proper threshold for the predicted probability.

### 3.6. Data Interpretation Using Imputed Datasets

A total of 308–354 proteins were experimentally identified for dataset 1, and 986–1001 proteins were experimentally identified for dataset 2, which are slightly over 10–30% of the whole proteome in *D. vulgaris*. Through the ANN modeling and imputation by integrating transcriptomic and proteomic data, we were able to assign abundance values to approximately 33–3400 proteins (dependent on growth conditions), approximately 96–98% of the whole proteomes in* D. vulgaris*. Using the imputed datasets, we first analyzed the proteins involved in energy metabolism when *D. vulgaris* was grown in three cultivation conditions (LL: Lactate-based growth in Exponential phase; LS: Lactate-based growth at Stationary phase; FL: Formate-based growth at Exponential phase). As shown in Table 4, even for proteins involved in energy metabolism such as ATP synthase which are typically expressed at relatively high levels, significant missing proteomic data was still obvious in the experimental datasets, only two out of nine putative ATP synthase proteins were experimentally detected under three growth conditions. By analysis of the imputed protein abundance for all putative ATP synthase proteins, a clear trend of downregulation was observed for LS and FL condition when compared with LL condition, which is consistent with the previous data suggesting lactate is a favorable electron donor for *D. vulgaris* [22]. When comparing lactate-based growth with formate-based at the exponential phase, we found that enzymes involved for formate utilization were predicted to be upregulated during growth on formate (Table 4). It has been reported that c-type cytochromes are highly expressed in sulphate-reducing bacteria [20], however, only low amounts of c-type cytochrome proteins have been identified from *D. vulgaris* by the proteomic (Table 4), probably due to the fact that c-type cytochromes undergo a complex posttranslational maturation process involving covalent attachment of heme groups [40]. Through the ANN model, significant expression of various cytochrome c proteins was predicted in *D. vulgaris* grown under three conditions. In general, cytochrome c proteins were predicted to have higher expression at the exponential phase in both lactate- and formate-based media than the stationary phase, consistent with their important roles in electron transfer during fast growth [20]. Two cytoplasmic hydrogenases (Ech and Coo) were previously assigned the putative function of generating hydrogen in the cytoplasm [20]. In agreement with previous prediction from the ZIP model [18], the ANN model predicted that all subunits of Ech hydrogenase (EchACDE) were upregulated, while all subunits of Coo hydrogenase (CooHLUX) were down-regulated on formate-based growth, suggesting their differential roles in H_2_ generation when grown with different carbon sources. We also used the imputed data to analysis the stress responses when the *D. vulgaris* cells were treated under salt stress. The analysis allowed identification of some proteins which were not experimentally detected, however, were predicted to be upregulated under the salt treatment condition (ST120) as compared with control (CT120) (Table 5). Among them were transporters and various multidrug resistance proteins and some signal transduction proteins. This list of proteins can serve as putative candidates for further experimental validation. In general, our analysis indicates that biological interpretation may benefit from imputing missing data using computational methods by integrating temporal transcriptomic and proteomic data. The full list of all protein-abundance predictions for all proteins has been provided in the Table  2 of the supplementary material. 

In conclusion, missing values in large-scale proteomic analysis is a frequent problem and in some cases it has created difficulty for accurate interpretation of proteomic data for complex biological systems. However, in spite of its obvious importance, the methodologies to deal with missing proteomic data are still lagging well behind the methods for transcriptomic analysis. In this study, we applied an artificial neural network approach to approximate the relationships between cognate transcriptomic and proteomic datasets of *D. vulgaris*, and to predict protein abundance for the proteins not experimentally detected based on relevant predictors, such as mRNA abundance, cellular role, and triple codon counts. The results showed that the coefficients of determination for the trained neural network models ranged from 0.47 to 0.68, providing better description than several previous models [18, 19]. In an attempt to identify possible causes for missing proteomic values, a logistic regression analysis of various experimental measurements and sequence-based features was performed for the experimentally undetected proteins but with high predicted protein abundance values. Although the results are in general consistent with previous knowledge that proteins with low possible expressivity and high instability will have higher chance to be missed, in the study, we for the first time quantified the odds ratio of each of those possible factors in the *D. vulgaris* datasets. In addition, in a preliminary analysis we demonstrated that biological interpretation could be improved by using the imputed proteomic datasets. Finally, although the model was well validated statistically, caution needs to be exercised when interpreting experimental data based on predicted protein expression values because the predicted abundance values are constrained by the quality of experimental proteomic data used as input, and a dearth of large-scale quantitative predictors which can be included in the model. Nevertheless, the initial success in applying the ANN method is encouraging, and the model could serve as a basis for developing more sophisticated models to further improve the biology interpretation.

## Supplementary Material

Supplementary Table 1: Relative variable importance measures for the ten top ranked variables (after removing genes with high mRNA values) (From Torres-García et al., *Bioinformatics*, 2009, 25:1905–1914).
Supplementary Table 2: Predicted values for all genes in different conditions.

## Figures and Tables

**Table 1:** Model validation: correlated expressions of proteins in selective operons.

	Dataset 1						Dataset 2					
	FL		LL		LS		CT0		CT120		ST120	
Operon	CV	PCV	CV	PCV	CV	PCV	CV	PCV	CV	PCV	CV	PCV
1	0.36	0.707	0.6	0.797	0.301	0.901	1.63*	1.248	0.814	1.186	1.269	1.286
2	1.6*	0.638	1.564*	0.725	1.48*	0.631	0.773	1.102	0.968	1.044	0.702	1.129
3	0.499	0.659	1.04*	0.766	0.563	0.866	1.295*	1.134	1.568*	1.106	1.324*	1.169
4	0.216	0.648	0.239	0.726	0.323	0.813	0.333	1.106	0.401	1.05	0.453	1.135
5	0.418	0.646	0.559	0.731	0.45	0.794	0.481	1.105	0.398	1.05	0.466	1.122
6	0.18	0.647	0.454	0.745	0.353	0.809	0.495	1.116	0.403	1.042	0.473	1.124
7	0.297	0.651	0.162	0.729	0.312	0.8	0.408	1.101	0.343	1.042	0.69	1.118
8	0.307	0.693	0.292	0.772	0.28	0.908	0.767	1.204	0.502	1.147	0.57	1.236
9	0.389	0.676	0.352	0.764	0.851	0.86	0.884	1.171	0.631	1.098	0.558	1.197
10	0.621	0.624	0.445	0.765	0.74	0.859	0.889	1.166	0.67	1.096	1.087	1.19
11	0.304	0.595	0.231	0.704	0.3	0.773	0.78	1.153	0.816	1.082	0.567	1.182
12	0.599	0.808	0.831	0.908	0.729	1.076	1.201	1.535	1.174	1.366	1.563*	1.528
13	0.353	0.646	0.435	0.723	0.531	0.826	0.366	1.118	0.532	1.06	0.369	1.124
14	0.359	0.665	0.407	0.73	0.267	0.752	0.653	1.157	0.825	1.087	0.597	1.18
15	0.381	0.717	0.707	0.801	0.78	0.922	0.691	1.246	0.838	1.166	0.674	1.293
16	0.299	0.749	0.538	0.833	0.307	0.989	0.613	1.342	0.456	1.252	1.059	1.372
17	0.321	0.649	0.219	0.726	0.222	0.806	0.657	1.059	0.492	0.943	0.529	1.046
18	0.473	0.645	0.486	0.723	0.628	0.767	0.474	1.164	0.539	1.091	0.578	1.185
19	0.592	0.741	0.479	0.824	0.631	0.982	1.68*	1.317	1.094	1.248	1.403*	1.342
20	0.767*	0.621	0.586	0.695	0.561	0.752	0.267	1.037	0.288	0.964	0.492	1.053
21	0.389	0.548	0.416	0.693	0.472	0.757	1.034	1.037	0.813	0.978	0.823	1.061
22	0.256	0.654	0.433	0.721	0.49	0.8	0.532	1.095	0.602	1.053	0.569	1.123
23	0.631	0.694	0.707	0.775	0.632	0.877	0.86	1.196	0.677	1.125	0.925	1.242
24	0.376	0.655	0.648	0.655	0.406	0.699	1.23*	1.099	0.927	1.031	1.138*	1.123
25	0.869*	0.674	1*	0.754	1.194*	0.85	2.337*	1.156	2.119*	1.094	2.303*	1.185
26	0.657*	0.618	0.683	0.736	0.747	0.856	0.696	1.151	0.52	1.114	0.7	1.176
27	0.378	0.85	0.452	0.938	0.432	1.104	0.588	1.631	0.667	1.445	0.771	1.629
28	1.013*	0.67	0.963*	0.764	1.526*	0.913	1.255	1.259	1.375*	1.181	1.332*	1.282

CV is computed by dividing SD by the mean of the prediction values for protein abundance for a specific set of genes (group). The protein prediction values were normalized by molecular weight before CV calculation. PCV is the mean of CV values computed through permutation test for selected operons.

*CV values greater than PCV.

**Table 2:** Estimates of odds ratios of missing proteomic values for dataset 1.

Dataset 1	LL			FL			LS		
effect	Point estimate	95% wald confidence limits		Point estimate	95% wald confidence limits		Point estimate	95% wald confidence limits	
log2 of mRNA	0.458	0.405	0.517	0.442	0.392	0.499	0.405	0.348	0.471
Protein instability index/10	1.628	1.379	1.922	1.493	1.282	1.739	1.539	1.297	1.825
log2 of gene length	0.477	0.373	0.608	0.517	0.415	0.644	0.419	0.324	0.543
*Nc*/10	0.465	0.271	0.798	0.446	0.272	0.732	0.314	0.174	0.566
CAI∗10	0.295	0.212	0.410	0.275	0.204	0.370	0.222	0.155	0.319
AA_axis2∗10	1.368	1.193	1.569	1.326	1.170	1.502	1.323	1.166	1.501
CR_axis3∗10	1.264	1.046	1.528	1.309	1.112	1.541	1.327	1.092	1.613
N_MFE∗10	—	—	—	—	—	—	1.880	1.178	3.000
AliphaticIndex/10	1.140	1.013	1.282	1.160	1.040	1.294	—	—	—

**Table 3:** Estimates of odds ratio of missing proteomic values for dataset 2.

Dataset 2	CT0			CT120			ST120		
effect	Point estimate	95% wald confidence limits		Point estimate	95% wald confidence limits		Point estimate	95% wald confidence limits	
log2 of mRNA	0.407	0.370	0.448	0.451	0.412	0.494	0.499	0.459	0.542
Protein instability index/10	1.310	1.161	1.478	1.283	1.140	1.444	1.264	1.124	1.421
log2 of gene length	0.261	0.216	0.315	0.287	0.240	0.345	0.294	0.247	0.352
*Nc*/10	0.512	0.336	0.780	0.518	0.343	0.783	0.552	0.374	0.815
GRAVY∗10	0.914	0.862	0.970	0.914	0.863	0.968	0.923	0.873	0.977
CAI∗10	0.599	0.421	0.851	0.559	0.398	0.786	0.536	0.384	0.749
AA_axis1∗10	0.686	0.627	0.750	0.681	0.624	0.744	0.686	0.629	0.749
AA_axis2∗10	1.811	1.558	2.106	1.721	1.487	1.990	1.674	1.452	1.931
AA_axis4∗10	0.827	0.747	0.916	0.837	0.757	0.925	0.846	0.767	0.934
CR_axis1∗10	0.771	0.647	0.919	0.780	0.657	0.925	0.781	0.660	0.923
CR_axis4∗10	0.716	0.635	0.808	0.706	0.627	0.795	0.725	0.645	0.814

**Table 4:** Regulation of proteins involved in energy metabolism for Dataset 1*.

Gene_ID	Protein abundance-Predicted-LL condition	RNA abundance-Measured-LL condition	Protein abundance-Measured-LL condition	Protein abundance-Predicted-LS condition	RNA abundance-Measured-LS condition	Protein abundance-Measured-LS condition	Protein abundance-Predicted-FL condition	RNA abundance-Measured-FL condition	Protein abundance-Measured-FL condition	Description
DVU0918	10.08	2315.20	x	2.46	416.75	x	5.71	1668.90	x	ATP synthase F0, A subunit (*atpB*)
DVU0779	6.55	2505.40	x	3.30	497.75	x	5.08	1397.10	x	ATP synthase F0, B subunit
DVU0780	6.62	3156.20	x	3.88	1050.10	x	5.81	2352.70	x	ATP synthase F0, B subunit
DVU0917	7.60	6086.50	x	3.85	1234.90	x	10.85	5944.40	x	ATP synthase F0, C subunit (*atpE*)
DVU0920	3.80	1590.50	x	3.59	947.88	x	5.75	1198.20	x	ATP synthase protein I (*atpI*)
DVU0777	39.24	2535.00	20.00	8.40	369.95	8.00	17.76	735.68	17.33	ATP synthase, F1 alpha subunit (*atpA*)
DVU0775	50.11	2228.60	49.33	23.76	711.53	30.33	39.80	1198.40	38.33	ATP synthase, F1 beta subunit (*atpD*)
DVU0778	4.25	1974.50	x	2.71	374.22	x	3.21	714.57	x	ATP synthase, F1 delta subunit (*atpH*)
DVU0774	4.85	2043.70	x	2.32	336.55	x	4.14	1113.40	x	ATP synthase, F1 epsilon subunit (*atpC*)
DVU0702	2.62	288.72	x	2.20	372.82	x	2.05	417.68	1.00	Cytochrome c family protein
DVU0922	1.69	66.60	x	1.85	149.38	x	1.49	149.52	x	Cytochrome c family protein
DVU1288	3.44	1172.20	x	2.06	298.10	x	3.12	826.90	x	Cytochrome c family protein
DVU2483	10.24	456.23	x	3.24	284.22	x	7.36	490.33	x	Cytochrome c family protein
DVU2484	5.06	575.50	x	2.39	277.35	x	4.63	665.95	x	Cytochrome c family protein
DVU2791	4.77	683.65	x	2.61	530.00	x	3.32	667.05	x	Cytochrome c family protein
DVU3107	15.10	1009.30	2.33	2.98	177.15	1.67	13.04	1026.30	3.00	Cytochrome c family protein
DVU3144	5.58	297.85	x	3.58	142.45	x	8.24	342.52	x	Cytochrome c family protein
DVU0625	30.20	1822.80	x	14.80	764.85	x	16.61	2467.50	x	Cytochrome c nitrite reductase, catalytic subunit NfrA
DVU1815	4.91	439.87	x	2.98	324.93	x	7.12	833.88	x	Cytochrome c oxidase, subunit I
DVU1812	4.42	304.57	x	2.72	215.70	x	4.63	335.70	x	Cytochrome c oxidase, subunit II
DVU1814	2.53	397.35	x	2.31	283.90	x	3.80	898.67	x	Cytochrome c oxidase, subunit III
DVU2809	3.11	447.42	x	2.32	395.15	x	2.10	601.50	x	Cytochrome c3
DVU3171	13.24	3937.00	3.33	5.24	966.25	x	11.82	5719.20	1.00	Cytochrome c3
DVU2524	1.77	114.95	x	2.08	135.07	x	1.61	170.70	x	Cytochrome c3,
DVU3041	3.03	894.60	x	2.62	713.10	x	2.73	684.27	x	Cytochrome c553
DVU1817	6.34	2280.00	11.67	7.26	1858.20	3.33	9.61	5400.20	12.00	Cytochrome c-553 (*cyf*)
DVU1048	2.31	255.07	x	2.14	243.58	x	1.53	185.47	x	Cytochrome c-type biogenesis protein CcmB (*ccmB*)
DVU1047	2.87	435.45	x	2.06	284.87	x	2.23	259.25	x	Cytochrome c-type biogenesis protein CcmC (*ccmC*)
DVU1051	3.30	915.92	x	3.59	769.45	x	4.19	732.07	x	Cytochrome c-type biogenesis protein CcmE (*ccmE*)
DVU1050	7.42	581.02	x	4.03	408.20	x	6.27	463.25	x	Cytochrome c-type biogenesis protein CcmF (*ccmF*)
DVU3271	35.79	8217.80	x	12.15	2295.10	x	11.38	6954.00	x	Cytochrome d ubiquinol oxidase, subunit I (*cydA*)
DVU3270	18.09	3877.70	x	5.50	1145.70	x	13.90	3897.10	x	Cytochrome d ubiquinol oxidase, subunit II (*cydB*)
DVU0434	1.28	177.22	x	2.79	168.72	x	5.13	468.00	x	Ech hydrogenase, subunit EchA
DVU0433	2.10	217.82	x	2.34	166.95	x	2.84	422.28	x	Ech hydrogenase, subunit EchB
DVU0432	2.11	222.90	x	2.24	133.13	x	3.12	595.60	x	Ech hydrogenase, subunit EchC
DVU0431	2.20	303.27	x	2.03	215.73	x	3.30	778.70	x	Ech hydrogenase, subunit EchD
DVU0430	2.68	259.00	x	2.52	199.20	x	4.61	685.78	x	Ech hydrogenase, subunit EchE
DVU0429	2.24	239.03	x	2.07	201.57	x	2.05	405.30	x	Ech hydrogenase, subunit EchF
DVU2824	0.90	124.20	x	3.54	106.78	x	9.30	102.10	x	Formate acetyltransferase
DVU2272	1.05	97.08	x	3.69	113.33	1.00	16.21	160.88	x	Formate acetyltransferase
DVU0578	2.11	154.12	x	2.23	217.48	x	2.03	247.52	x	Formate dehydrogenase accessory protein FdhD
DVU0577	2.56	237.93	x	2.29	148.02	x	1.97	513.40	x	Formate dehydrogenase formation protein FdhE
DVU2810	3.39	423.00	x	2.27	312.02	x	2.47	510.12	x	Formate dehydrogenase formation protein FdhE
DVU0588	0.92	35.70	x	2.65	35.25	x	1.26	79.03	x	Formate dehydrogenase, beta subunit
DVU2481	2.39	227.43	x	2.37	304.92	x	2.35	399.82	x	Formate dehydrogenase, beta subunit
DVU2811	3.22	335.33	x	2.52	181.45	x	3.13	435.95	x	Formate dehydrogenase, beta subunit
DVU2288	2.68	781.45	x	2.24	412.85	x	1.39	139.17	x	Hydrogenase, CooL subunit
DVU2286	16.67	308.33	x	3.29	138.72	x	6.08	81.55	x	Hydrogenase, CooM subunit
DVU2290	2.84	703.22	3.00	2.10	316.45	3.00			x	Hydrogenase, CooU subunit
DVU2289	3.92	823.18	x	2.14	553.33	x	2.36	319.80	x	Hydrogenase, CooX subunit

*Protein abundance is in peptide counts; RNA abundance is in raw fluorescence measurements in microarrays. “x” indicated proteins are undetected experimentally.

**Table 5:** Up-regulated proteins under stress condition for Dataset 2*.

Gene_ID	Protein abundance-Predicted-CT120	RNA abundance-Measured-CT120	Protein abundance-Measured-CT120	Protein abundance-Predicted-ST120	RNA abundance-Measured-ST120	Protein abundance-Measured-ST120	Description
*Amino acid biosynthesis*							

DVU0285	5.00	3005.10	x	7.43	4051.50	x	Imidazole glycerol phosphate synthase (hisH)
DVU1665	21.82	20264.00	x	50.55	30766.00	x	3-dehydroquinate dehydratase, type II (*aroQ*)

*Biosynthesis of cofactors, prosthetic groups, and carriers*							

DVU0157	5.45	7559.00	x	6.31	5092.30	x	Thiamin-monophosphate kinase (*thiL*)
DVU0931	5.41	1730.40	x	6.87	4401.40	x	Phosphomethylpyrimidine kinase (*thiD*)
DVU1200	5.82	5439.20	x	6.75	5079.60	x	Riboflavin synthase, alpha subunit (*ribE*)
DVU2749	2.85	1427.30	x	3.59	856.35	x	Precorrin-6Y C5,15-methyltransferase (decarboxylating) (*cobL*)
DVU3307	14.65	20224.00	x	26.42	30616.00	x	3-octaprenyl-4-hydroxybenzoate carboxy-lyase (*ubiX*)

*Cell envelope*							

DVU0294	2.44	1034.50	x	3.15	726.82	x	Glycosyl transferase, group 2 family protein
DVU0308	2.97	1552.00	x	7.93	6986.70	x	Membrane protein
DVU0328	8.59	5628.30	x	9.12	5042.40	x	Glycosyl transferase, group 1 family protein
DVU0407	26.65	20237.00	x	38.70	32568.00	x	Rare lipoprotein A family protein
DVU1534	2.48	1792.60	x	3.56	2183.30	x	Membrane protein
DVU2053	2.98	2406.60	x	3.85	2507.60	x	Membrane protein
DVU2102	2.91	628.30	x	3.88	843.26	x	Outer membrane protein, OMP85 family
DVU2356	20.54	6021.30	x	36.26	7883.90	x	Membrane protein
DVU2672	3.30	3253.30	x	4.47	5875.90	x	Membrane protein
DVU2725	5.20	4925.80	x	13.97	8894.70	x	Membrane protein

*Cellular processes*							

DVU0302	28.35	22358.00	x	37.21	21180.00	x	Chemotaxis protein CheX
DVU2069	2.18	811.21	x	3.72	658.60	x	DNA processing protein DprA
DVU2816	4.82	539.18	x	8.65	1502.80	x	Multidrug resistance protein
DVU2893	4.59	9413.30	x	6.78	9241.60	x	Flagellar basal-body rod protein
DVU3326	3.13	782.67	x	3.68	2732.60	x	Multidrug resistance protein, Smr family
DVUA0002	5.53	2257.50	x	12.96	6384.00	x	ParA family protein
*DNA metabolism*							

DVU0878	3.20	1967.30	x	7.13	11149.00	x	dnaK suppressor protein
DVU1353	2.26	1516.00	x	10.31	868.27	x	DNA polymerase III, alpha subunit (*dnaE*)
DVU1703	1.93	906.31	x	2.69	628.53	x	Type I restriction-modification enzyme, R subunit

*Energy metabolism*							

DVU0080	4.42	2476.60	x	6.45	5175.70	x	Fumarate hydratase, class II (*fumC*)
DVU0531	6.57	328.82	x	11.02	1157.90	x	Hmc operon protein 6
DVU2137	3.64	389.78	x	4.45	338.82	x	Succinyl-CoA synthase, beta/alpha subunits (*sucCD*)

*Other categories*							

DVU0197	3.46	933.13	x	4.40	285.54	x	Phage portal protein, lambda family
DVU1490	4.72	351.64	x	5.70	372.18	x	Tail tape measure protein

*Regulatory functions*							

DVU0063	3.74	3823.20	x	4.79	6404.70	x	Transcriptional regulator, MarR family
DVU1331	7.54	6812.50	x	8.33	11619.00	x	Transcriptional regulator, LysR family
DVU1645	7.22	7075.60	x	14.03	23482.00	x	Transcriptional regulator, ArsR family
DVU3062	2.34	1205.40	x	2.92	570.26	x	Sensor histidine kinase/response regulator
DVU3095	12.30	17923.00	x	14.86	23528.00	x	Transcriptional regulator, Fur family
DVU0721	4.20	1085.90	x	7.19	513.28	x	Sensory box histidine kinase
DVU0834	5.73	2795.50	x	12.17	13860.00	x	Ribonuclease HII (rnhB)
DVU1075	13.63	30772.00	x	15.45	27576.00	x	Ribonuclease P protein component (rnpA)

*Transport and binding proteins*							

DVU0593	2.58	992.23	x	3.28	1151.30	x	L-lysine exporter
DVU1068	3.92	301.98	x	5.76	270.52	x	Branched-chain amino acid ABC transporter, permease proteins
DVU2340	6.00	11276.00	x	7.00	11625.00	x	Amino acid ABC transporter, permease protein
DVU2572	3.19	2831.50	x	5.77	7937.50	x	Ferrous iron transport protein A
DVU2574	4.62	5005.50	x	8.85	8857.00	x	Ferrous ion transport protein
DVU2744	17.96	1201.00	x	34.42	3053.50	x	High-affinity branched-chain amino acid ABC transporter
DVUA0136	3.29	673.57	x	3.89	346.83	x	Zinc transporter (*zupT*)

*Unknown function*							

DVU0155	8.35	16519.00	x	12.15	18298.00	x	Type I phosphodiesterase/nucleotide pyrophosphatase family proteins
DVU0938	10.12	15409.00	x	11.76	13138.00	x	Isoamylase N-terminal domain protein
DVU1023	7.26	123.66	x	8.15	144.26	x	Rhomboid family protein
DVU1202	5.39	3480.30	x	5.92	5395.40	x	Cytidine/deoxycytidylate deaminase family protein
DVU1558	5.77	189.07	x	6.47	234.72	x	Cysteine-rich domain/iron-sulfur cluster-binding domain proteins
DVU1730	3.16	1288.70	x	3.74	4503.10	x	DNA-binding protein
DVU1745	6.55	319.33	x	7.58	182.38	x	DNA-binding protein
DVU1747	5.08	258.33	x	12.80	230.72	x	ATPase, histidine kinase-, DNA gyrase B-, and HSP90-like domainsprotein
DVU2036	4.27	1086.70	x	5.07	350.61	x	Helix-turn-helix protein, CopG family
DVUA0039	3.75	269.47	x	4.27	261.42	x	Chain length determinant family protein

*Protein abundance is in peptide counts; RNA abundance is in raw fluorescence measurements in microarrays. “x” indicated proteins are undetected experimentally.
